# The Role of Maternal Gut Firmicutes/Bacteroidetes Ratio in Shaping Fetal Development and Neonatal Microbial Communities

**DOI:** 10.3390/life15070990

**Published:** 2025-06-20

**Authors:** Arianna Omaña-Covarrubias, Luis Guillermo González-Olivares, Lydia López Póntigo, Ana Teresa Nez-Castro, Rogelio Cruz-Martínez, Marcela Hernández-Ortega

**Affiliations:** 1 Research Center for Health Sciences (CICSA), Faculty of Health Sciences, Universidad Anáhuac México, Huixquilucan 52786, Mexico; aomana@uaeh.edu.mx; 2 Academic Area of Nutrition, Institute of Health Sciences, Universidad Autónoma del Estado de Hidalgo, San Agustín Tlaxiaca 42060, Mexico; teresa_nez@uaeh.edu.mx; 3 Academic Area of Food Chemistry, Institute of Basic Sciences and Engineering, Universidad Autónoma del Estado de Hidalgo, San Agustín Tlaxiaca 42060, Mexico; lgonzales@uaeh.edu.mx; 4 Academic Area of Gerontology, Institute of Health Sciences, Universidad Autónoma del Estado de Hidalgo, San Agustín Tlaxiaca 42060, Mexico; lydial@uaeh.edu.mx; 5 Fetal Medicine Department, Instituto Medicina Fetal México, Children and Women’s Specialty Hospital of Querétaro, Querétaro 76090, Mexico; rcruz@medicinafetalmexico.com

**Keywords:** gut microbiota, pregnancy, fetal development, newborn

## Abstract

According to research, intrauterine exposure to non-pathogenic maternal microorganisms during pregnancy is influenced by the mother’s nutritional, metabolic, and immunological status. This study investigates the association between maternal gut microbiota composition, fetal development, and neonatal microbiota, with the aim of exploring their interconnected health dynamics. A cohort-based correlational study was conducted involving 114 women (≥18 years old, ≤12 weeks of gestation) attending prenatal consultations at the ISSSTE General Hospital in Pachuca de Soto, Hidalgo, México. Data were collected at four stages: before 11 weeks, at 11–14 weeks, at 20–24 weeks, and at 31 weeks of pregnancy. Assessments included anthropometric measurements, biochemical markers, and intestinal microbiota analysis. The Firmicutes/Bacteroidetes (F/B) ratio positively correlated with venous duct flow and expected weight for gestational week (r = 0.02272, *p* = 0.0323; r = 0.2344, *p* = 0.0271). Bacteroidetes showed a positive correlation with birth weight (r = 0.2876, *p* = 0.0063), birth height (r = 0.5889, *p* < 0.001), and head circumference (r = 0.2163, *p* = 0.0418). Correlation analysis revealed significant relationships between maternal and neonatal microbiota, particularly for Bacteroidetes and Firmicutes. The findings suggest that maternal gut microbiota significantly influences fetal growth and neonatal microbiota composition. These insights underscore the importance of maternal health during pregnancy.

## 1. Introduction

The mother’s condition during pregnancy directly affects the fetus’s nutritional status and growth, influencing development at birth [1]. Maternal factors are crucial, as any dysfunction cannot only impair uterine growth but also disrupt fetal genetic programming. This may lead to functional alterations in the fetus, increasing the risk of diseases that manifest later in childhood, adolescence, or adulthood [2].

Pregnancy is a biological process characterized by metabolic, endocrine, and immunological changes, such as metabolic syndrome. These include weight gain, elevated blood glucose levels, insulin resistance, alterations in metabolic hormones, and shifts in gut microbiota composition [3].

The intrauterine environment was once considered sterile, with microbial colonization believed to begin only after birth. However, DNA from gastrointestinal bacteria has been detected in the placenta, umbilical cord, amniotic fluid, and meconium, suggesting that maternal bacteria can be transferred to the fetus during the perinatal period [4].

Recent research suggests that bacterial colonization begins in utero, programmed through the placenta and amniotic fluid, with significant colonization occurring at birth. Intrauterine exposure to non-pathogenic maternal microorganisms during pregnancy is now understood to be a dynamic process influenced by the mother’s nutritional, metabolic, and immunological status throughout pregnancy [5].

Numerous studies highlight the critical role of gut microbiota in nutrition and health, emphasizing its interaction with diseases, particularly non-communicable ones, where chronic inflammation contributes to the development of dysbiosis. The most representative bacteria in the colon belong to three major phyla: *Firmicutes* (40–60%), *Bacteroidetes* (20–40%), and *Actinobacteria* (>10%) [6].

In obesity, a reduction of approximately 50% in the Bacteroidetes phylum has been observed, which is associated with an increase in high-density lipoproteins (HDL-c) and folic acid [7]. Furthermore, this phylum is involved in activating the immune system by stimulating T cell-mediated responses and controlling the colonization of pathogenic bacteria that utilize polysaccharides as substrates [8].

Moreover, *Firmicutes* are notable for their efficiency in extracting energy from insoluble fiber, which consequently contributes to weight gain due to the increased energy availability in peripheral cells that promotes the adipogenesis process [9].

This study aimed to examine the association between gut microbiota composition, body composition, and metabolic and clinical indicators in both the pregnant mother and the fetus. The results may provide evidence that pregnancy itself is a complex and dynamic process and plays a significant role in shaping microbial communities across different stages and individuals. Thus, data may inform early strategies to support maternal and neonatal health through microbiota-targeted approaches.

## 2. Materials and Methods

A correlational cohort study involved 89 women who participated in prenatal consultations at the ISSSTE General Hospital in Pachuca de Soto, Hidalgo. The sample size was calculated using Sigma Plot v12 software (Chicago, IL, USA) based on a correlation coefficient of 0.26, a statistical power of 0.8, and a type I error of 0.05. The study did not include a control group due to the specific inclusion criteria, which were as follows: participants had to be over 18 years old, had to have a maximum gestational age of 12 weeks, and had to have signed informed consent. The exclusion criteria included gestational age over 12 weeks, pregnancy interruption, and failure to attend prenatal check-up appointments. Additionally, the elimination criteria included withdrawal of informed consent and inability to undergo structural ultrasound evaluation.

Once the study population was determined, the pregnant women were considered as a mother-fetus-newborn unit and were evaluated at five time points: (1) before the 11th week of gestation; (2) between the 11th and 14th weeks of gestation; (3) between the 20th to 24th week of gestation; (4) after the 31st week of gestation, and (5) at birth.

The study received approval from the ethics committees of Anahuac University (202103) and ISSSTE Pachuca General Hospital (017-2022). It was conducted following the International Ethics Guidelines for Biomedical Research Involving Human Subjects, the Declaration of Helsinki, the Nuremberg Code, and NOM-012-SSA3-2012, which governs national regulations. The research adhered to the Regulations of the General Health Law on Research and was designed to involve minimal risk. Each participant was thoroughly informed about the procedures involved, and their decision to participate was documented through a signed informed consent form.

### 2.1. Anthropometric Evaluation

Patients attended monthly prenatal control appointments, where their weight was measured using an InBody 270 analyzer. Height was recorded with a portable SECA 213 stadiometer (Wandsbek/Eilbek, Hamburg, Germany). This was performed as follows: the patient stood with their back to the stadiometer, heels aligned with the longitudinal axes of both feet, maintaining a 45° angle between them, and keeping the head in the Frankfort plane. BMI (Body Mass Index) was then calculated using height and weight measurements.

Fetal growth was assessed at four stages: the first before 11 weeks of gestation, the second between weeks 11 and 14, the third from weeks 20 to 24, and the fourth after week 31. Ultrasound measurements included cranial-caudal length, head circumference, abdominal circumference, femur length, biparietal diameter, nuchal fold, venous duct flow, fetal heart rate, and estimated weight for the gestational week.

Finally, in the newborns, length was measured using a SECA^®^ 416 infantometer (Wandsbek/Eilbek, Hamburg, Germany). Head circumference was obtained with a SECA^®^ 212 headband (Wandsbek/Eilbek, Hamburg, Germany), and weight was recorded using a SECA^®^ 376 baby scale (Wandsbek/Eilbek, Hamburg, Germany).

### 2.2. Biochemical Samples

Pregnant women were instructed to fast for no more than 8 h before providing a blood sample. A vein in the inner elbow was selected and cleaned with an antiseptic solution. An elastic band was applied around the upper arm, and a needle was inserted into the vein to collect the sample. After the sample was obtained, the elastic band and needle were removed, and a bandage was applied to stop the bleeding. The collected samples were then transported to a clinical laboratory for spectrophotometry analysis.

### 2.3. Blood Pressure Measurement

Blood pressure was measured for pregnant women during each prenatal check-up visit. The women were seated with their backs against the chair, legs uncrossed, and feet flat on the floor, with their forearms at heart level. A sphygmomanometer was comfortably positioned around the upper arm. The bulb was pumped while the cuff valve was gradually opened to allow the pressure to decrease slowly. As the pressure fell, the systolic reading was noted when the blood flow sound was first heard, and the diastolic pressure was recorded when the sound disappeared.

### 2.4. Fetal Ultrasound

The ultrasonography test was conducted in four stages. The pregnant women were positioned on the examination bed, and their abdomens were exposed. An appropriate wavelength ultrasound gel was applied, and the transducer was used to locate the areas of interest for the study.

### 2.5. Analysis of Intestinal Microbiota

Fecal Sample Collection. Fecal samples from the mother during the first and third trimesters were collected. The samples during the first trimester were collected from the first bowel movement on the day of the evaluation. The third-trimester samples were collected from the first postpartum bowel movement. For the meconium samples, some were collected at birth and others during the first bowel movement in the diaper. All the samples were frozen at −80 °C for subsequent analysis.

DNA extraction. The extraction was performed using the QIAamp DNA Stool Kit (Markham, ON, Canada), following the manufacturer’s instructions. The concentration and purity of the extracted DNA were determined by spectrophotometry using the Nanodrop-2000c spectrophotometer (Wilminton, DE, USA).

DNA amplification. The quantifications of *Bacteroides fragilis* and *Firmicutes* spp. were conducted using PCR-RT with the following *Firmicutes* and *Bacteroidetes* primers, according to Martínez et al. [10].

Bacteroidetes: 798CFBf AAACTCAAAKGAATTGACGG (Forward) and cfb967R GGTAAGGTTCCTCGCGCTAT (Reverse).

Firmicutes: 928F-firm TGAAACTYAAGGAATTGACG (Forward) and 1040fIRMr ACCATGCACCACCCTGTC (Reverse).

Quantification of Intestinal Microbiota. Bacterial quantities were determined by volume using standard curves created from serial dilutions ranging from 10^2^ to 10^6^ based on the McFarland 4 scale, including control strains for each bacterium.

### 2.6. Statistical Analysis

The Kolmogorov–Smirnov test was conducted to assess the normality of the data. To analyze differences between measurement cohorts, the Wilcoxon rank-sum test was applied to the variables of Bacteroidetes and Firmicutes and their ratio in the mothers, along with crown-rump length, venous duct flow, and estimated weight for gestational age in fetuses.

Additionally, Friedman’s post hoc Dunn’s test was performed on maternal variables such as weight, BMI, glucose, total cholesterol, triglycerides, and both systolic and diastolic blood pressure, as well as fetal variables including biparietal diameter, head circumference, abdominal circumference, femur length, and fetal heart rate.

The Mann–Whitney U test was used to compare maternal and fetal microbiota. In contrast, associations between the mother’s microbiota and the fetus’s microbiota were examined using Spearman correlation, considering a *p* < 0.05. All analyses were conducted using SPSS statistical software, version 21.

## 3. Results

A total of 89 mother-fetus-newborn pairs were evaluated during the period from August 2022 to May 2023.

### 3.1. Maternal Anthropometric, Biochemical, and Microbiota Changes During Pregnancy

Table 1 presents the results based on maternal variables. According to the anthropometric indicators, the most frequent Body Mass Index (BMI) at the start of the evaluation was 26.5 kg/m^2^, suggesting that the sample of pregnant women began their pregnancies classified as overweight. Significant differences were observed at each consultation, even when taking into account the expected weight gain. On average, the women gained 14 kg during the study, which led to an average BMI categorized as obesity grade I (BMI = 30.9, 28.9–33.9).

Regarding the biochemical indicators, glucose levels initially remained within normal ranges. However, after analyzing the interquartile ranges, hyperglycemia was observed starting from the fourth measurement. A similar pattern was observed in the lipid profile, with statistically significant differences detected beginning from the third measurement. Despite these variations, lipid levels remained normal throughout all measurement periods.

In terms of the intestinal microbiota characterization, the balance of its composition was initially maintained. Nevertheless, significant differences were observed between the first and last measurements, particularly in the Firmicutes/Bacteroidetes (F/B) ratio. An increase in the abundance of Firmicutes was identified over time.

### 3.2. Fetal and Newborn Variables

The results in Table 2 present the fetal variables with significant differences in each measurement, indicating appropriate growth. Among the newborns, 61.8% were delivered by cesarean section, while 38.2% were delivered vaginally. Analysis of newborn variables revealed that the average weight, height, and head circumference were consistent with those expected for full-term infants.

Additionally, the ratio of Bacteroidetes to Firmicutes suggests a balanced microbiome, with Bacteroidetes predominating over Firmicutes, as detailed in Table 3.

The comparative analysis revealed no significant differences between the microbiota of mothers and their newborns. This suggests that the newborn’s microbiota shares essential similarities with the mother’s, as illustrated in Table 4.

**Figure 1:**
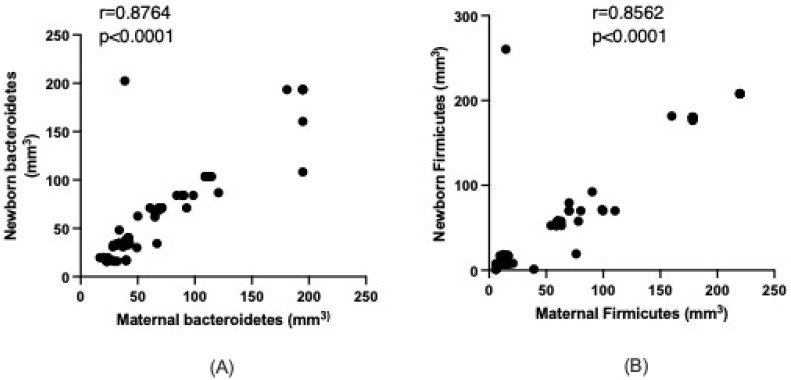
Relationship between maternal and newborn Bacteroidetes (**A**) and Firmicutes (**B**). Data analyzed with the Spearman test.

Given the observed similarity between the microbiota of mothers and newborns, we further analyzed the correlation between *Bacteroidetes* and *Firmicutes*. The results indicated a strong positive association with *Bacteroidetes* in the newborn positively correlating with the maternal intestinal microbiota (r = 0.8764, *p* < 0.0001). Similarly, a positive correlation was found for *Firmicutes* between the newborn and the mother (r = 0.8562, *p* < 0.0001), as depicted in Figure 1A,B.

### 3.3. Maternal–Newborn Microbiota Relations

Given the observed relationships between the maternal and newborn microbiota, associations were analyzed for *Bacteroidetes* and *Firmicutes*. Along with their ratios, various measurements were taken from the mother, fetus, and newborn. These associations are detailed in Table 5.

As observed, none of the maternal measurements correlated with the concentration of *Bacteroidetes*. However, maternal *Firmicutes* are positively associated with total cholesterol (r = 0.3676, *p* = 0.0004) and diastolic pressure (r = 0.2598, *p* = 0.0140). An inverse relationship was found between *Firmicutes* and c-HDL (r = −0.3302, *p* = 0.0016).

Regarding the *Firmicutes*/*Bacteroidetes* ratio, positive associations were noted with total cholesterol (r = 0.3490, *p* = 0.0008), triglycerides (r = 0.2137, *p* = 0.0444), and both systolic (r = 0.2999, *p* = 0.0043) and diastolic pressure. A negative correlation was also identified with c-HDL concentration (r = −0.3014, *p* = 0.0041).

Table 6 shows the correlation between maternal *Bacteroidetes* and various fetal measurements.

Notable correlations include cranial-rump length in the first (r = 0.4005, *p* = 0.0001) and second measurements (r = 0.3055, *p* = 0.0036). Additionally, a positive correlation was found in the third ultrasound for abdominal circumference (r = 0.2854, *p* = 0.0067), femur length (r = 0.3417, *p* = 0.0010), and expected weight for gestational age (r = 0.4921, *p* < 0.0001). These results highlight the significant associations between maternal *Bacteroidetes* and fetal growth parameters.

In the final check-up, a negative association was observed with certain variables that previously showed positive correlations in the third ultrasound, notably femur length (r = −0.2310, *p* = 0.0294). A positive correlation was found for *Firmicutes* with rump length in the first measurement (r = 0.2715, *p* = 0.0101) and head circumference in the third ultrasound (r = 0.2278, *p* = 0.0318). Additionally, *Firmicutes* demonstrated positive correlations with abdominal circumference and the venous duct across most ultrasound measurements.

Similarly to *Bacteroidetes*, a transition from positive to negative correlation was noted for expected weight based on gestational age, changing from r = 0.4653, *p* < 0.0001 to r = −0.2890, *p* = 0.0060. These findings indicate a complex relationship between maternal microbiota and fetal growth parameters throughout pregnancy.

A positive relationship was observed for the *Firmicutes*/*Bacteroidetes* ratio with the venous ductus (r = 0.2272, *p* = 0.0323) and expected weight for gestational age (r = 0.2344, *p* = 0.0271). A similar pattern was noted for *Bacteroidetes* regarding expected weight, where a positive correlation was found in the third ultrasound (r = 0.2710, *p* < 0.0001), transitioning to a negative correlation in the final ultrasound (r = −0.3635, *p* = 0.005).

Observing the newborn measurements, *Bacteroidetes* showed positive and significant correlations with several variables, including birth weight (r = 0.2876, *p* = 0.0063), birth height (r = 0.5889, *p* < 0.001), and head circumference at birth (r = 0.2163, *p* = 0.0418). These findings highlight the impact of maternal microbiota on newborn growth parameters.

## 4. Discussion

Pregnancy is a physiological state associated with changes in hormone levels, body structure, and modifications in metabolic, inflammatory, and immune status, all of which are closely related to embryo implantation and fetal growth. Several studies have focused on determining whether these changes during pregnancy are related to the gut microbiota, finding that taxonomic composition and diversity remain remarkably stable throughout pregnancy. This suggests that differences in metabolic, inflammatory, and immune responses—as well as the development of various pathologies—are directly related to the initial maternal-specific microbial taxa and the molecules they produce [11,12,13].

Since previous studies have focused only on maternal microbiome changes or on the fetal and newborn microbiome separately, the present study analyzed the relationship between the maternal microbiome and the fetal and newborn microbiota. The results indicate a close association between the mother’s health status and the newborn, as suggested by Taylor, offering a possible explanation through the concept of fetal programming as outlined by the fetal origin hypothesis. This hypothesis posits that those metabolic factors in the intrauterine environment, such as elevated levels of glycemia, triglycerides, and inflammatory cytokines, can have long-term effects on health.

For instance, early food restriction during pregnancy may not impact birth weight but can increase the risk of obesity, glucose intolerance, cardiovascular disease, and adverse lipid profiles in adulthood. Conversely, food restriction in mid-pregnancy can affect kidney function, glucose tolerance, and insulin secretion. Late or end-of-pregnancy restrictions typically result in low birth weight without an increased risk of obesity; however, they can still lead to glucose intolerance later in life [14,15]. These findings underscore the importance of maternal health in shaping the long-term outcomes of offspring.

For many years, it was believed that colonization of a baby’s intestines begins at birth. However, recent studies suggest that this process starts in the uterus, although the diversity of the microbiota is lower at this stage compared to later in life. This understanding aligns with the findings of this study, where the participating mothers’ intestinal composition exhibited similarities and positive correlations in terms of Firmicutes, Bacteroidetes, and their ratio [16]. This emphasizes the significance of maternal microbiota in shaping the early gut microbiome of infants.

Evidence supporting in utero colonization continues to grow, revealing similarities between the intestinal microbiota and the placenta. Maternal obesity significantly influences this colonization, primarily due to the increase in Firmicutes. In the mothers who participated in this study, the overweight status observed at the end of pregnancy was pronounced, resulting in a BMI classified as obese. This condition appeared to be inherited by the newborns, as no statistically significant differences were found in their outcomes.

This phenomenon was noted in women who gained more than 16 kg during pregnancy, and this study replicated similar results with an average weight gain of 14 kg. These findings underscore the potential impact of maternal obesity on both maternal and neonatal health [17].

Based on the above, understanding that metabolic processes may vary in pregnant women due to changes in the gut microbiota during pregnancy is important [18,19]. This highlights the importance of maintaining optimal health not only before conception but also throughout pregnancy in order to positively influence fetal growth and, more importantly, intrauterine colonization, thereby supporting healthy development from birth.

It is important to acknowledge the limitations of this study. One such limitation was the lack of dietary evaluation. Since the study was conducted during the COVID-19 pandemic, participants unconsciously changed their eating habits. Therefore, it is possible that some of the observed changes in microbiota composition were not solely due to pregnancy but also due to these dietary alterations.

Additionally, only weight gain was considered as an indicator of fetal growth. However, during pregnancy, there is an increase in extracellular fluid volume, including both plasma and interstitial fluid, which accounts for approximately 50% of total weight gain. Morphological changes also occur, such as enlargement of organs like the kidneys and uterus, which contribute to weight gain. Furthermore, hormonal and metabolic changes affect body composition compartments, such as fat and water percentages, which are not necessarily related to poor eating habits. These changes are driven by increased levels of hormones that include progesterone, placental lactogen, and estrogens, whose secretion progressively rises throughout pregnancy.

Finally, the analysis focused solely on the composition of Firmicutes and Bacteroidetes, as existing evidence suggests that the ratio between these bacterial phyla is associated with changes in energy metabolism and inflammatory processes, which are already altered during pregnancy. However, future research should consider including Proteobacteria and Actinobacteria, as these also play an important role in fetal growth, particularly when considering gestational age at birth.

## 5. Conclusions

During the evaluations, fetal growth was assessed as appropriate in relation to gestational weeks. All study variables showed statistical significance except for fetal heart rate, which remained consistent regardless of gestational age. When the intestinal microbiota of mothers and newborns were compared, no significant differences were found, indicating a similarity in microbiota composition between the two. Subsequent correlation analyses revealed a positive relationship between the maternal microbiota and that of the newborn, specifically for Bacteroidetes, Firmicutes, and the Firmicutes/Bacteroidetes ratio. These findings highlight the connection between maternal and neonatal microbiota.

Future research is recommended to revisit studies examining the intestinal microbiota in both mothers and fetuses, as well as its potential impacts on fetal and newborn development. Continued exploration of these variables could yield valuable insights into how microbiota influences the health outcomes of mothers and infants. This line of inquiry has the potential to inform interventions that promote healthier microbiota profiles, ultimately benefiting maternal and neonatal health. Moreover, it may help identify microbial markers associated with favorable outcomes, contributing to personalized maternal care and improved birth outcomes.

## Figures and Tables

**Table 1:** Characterization of the pregnant woman.

Parameter	Time 1	Time 2	Time 3	Time 4	Time 5	*p*
Height (m) Median (Q1–Q3)	1.60 (1.58–1.63)					
Weight, kg Median (Q1–Q3) *	68.6 (60.0–81.0) ^a^	71.0 (61.0–83.0) ^b^	73.0 (64.0–85.5) ^c^	77.0 (67.4–88.0) ^d^	82.0 (73.0–91.5) ^e^	<0.0001
BMI (kg/m^2^) Median (Q1–Q3) *	26.5 (24.1–30.1) ^a^	27.8( 24.8–30.9) ^b^	28.9 (25.5–31.7) ^c^	29.8 (27.2–32.7) ^d^	30.9 (28.9–33.9) ^e^	<0.0001
Glucose (mg/dL) Mediana (Q1–Q3) *	83.0 (76.0–93.0) ^a^	87.0 (78.0–95.0) ^b^	86.0 (78.0–94.5) ^b^	88.0 (80.0–98.5) ^c^	90.0 (84.0–100.0) ^c^	<0.0001
TC (mg/dL) Median (Q1–Q3) *	138.0 (108.5–167.0) ^a^	140.0 (111.0–162.5) ^a^	140.0 (114.0–163.0) ^b^	140.0 (115.0–160.0) ^b^	140.0 (116.0–180.0) ^b^	<0.0001
TG (mg/dL) Median (Q1–Q3) *	103.0 (90.0–128.0) ^a^	110.0 (90.0–125.0) ^a^	110.0 (91.0–118.0) ^b,a^	110 (91.0–129.0) ^a^	110 (95.0–133.0) ^a^	<0.0001
cHLD (mg/dL) Median (Q1–Q3) *	31.0 (26.5–39.0) ^a^	29.0 (25.0–37.0) ^a^	30.0 (25.0–38.5) ^a^	30.0 (21.5–37.5) ^a^	30.0 (20.0–36.0) ^a^	<0.0001
Systolic pressure (mmHg) Median (Q1–Q3) *	118.0 (115.0–120.0) ^a^	118.0 (110.0–119.5) ^a^	115.0 (110.0–121.5) ^a^	115.0 (110.0–120.0) ^a^	119.0 (110.0–124.0) ^a^	>0.9999
Diastolic pressure (mmHg) Median (Q1–Q3) *	78.0 (75.0–80.0) ^a^	77.0 (70.0–78.0) ^a^	76.0 (71.0–78.0) ^a^	77.0 (73.0–80.0) ^a^	77.0 (70.0–80.0) ^a^	0.1638
*Bacteroidetes* (mm^3^) Mediana (Q1–Q3) **	39.7 (32.1–72.6)				39.8 (33.0–69.8)	0.2720
*Firmicutes* (mm^3^) Median (Q1–Q3) **	13.1 (8.8–61.6)				15.4 (10.4–66.7)	<0.0001
Ratio F/B Median (Q1–Q3) **	0.38 (0.24–0.58)				0.44 (0.27–0.67)	<0.0001

*n* = 89. TC: total cholesterol, TG: triglycerides. Different superscript lowercase letters in the same row indicate the presence of statistically significant differences, *p* < 0.05. * Data was analyzed using the Friedman test and Dunn’s post hoc test. ** Data was analyzed with the Mann–Whitney U test.

**Table 2:** Characterization of the fetus.

Parameter	Time 1	Time 2	Time 3	Time 4	*p*
Crown-rump length (mm) Median (Q1–Q3) *	19.0 (11.0–24.0) ^a^	76.3 (70.0–90.4) ^b^			<0.0001
DBP (mm) Median (Q1–Q3) **		27.0 (25.2–33.8) ^a^	59.1 (53.9–62.3) ^b^	86.4 (80.1–88.8) ^c^	<0.0001
Nuchal translucency (mm) Median (Q1–Q3)		2.10 (1.47–2.34)			
Head circumference (mm) Median (Q1–Q3) **		94.1 (87.0–101.7) ^a^	210.2 (207.1–229.3) ^b^	298.0 (288.2–316.2) ^c^	<0.0001
Abdominal circumference (mm) Median (Q1–Q3) **		81.2 (75.7–119.7) ^a^	192.0 (184.1–203.9) ^b^	290.0 (273.6–311.6) ^c^	<0.0001
Femur length (mm) Median (Q1–Q3) **		13.0 (10.6–19.8) ^a^	43.1 (39.0–45.2) ^b^	63.1 (59.8–67.0) ^c^	<0.0001
Fetal heart rate (lpm) Median (Q1–Q3) **		149.0 (145.5–158.0) ^a,b^	156.0 (145.5–163.0) ^b,b^	153 (140–165) ^a,b^	0.013
Venous duct (mm) Median (Q1–Q3) *		1.03 (0.97–1.90) ^a^	0.78 (0.58–0.90) ^b^		<0.0001
Estimated weight (g) Median (Q1–Q3) *		483 (380–674) ^a^	2115 (1875–2507) ^b^		<0.0001

*n* = 89 Different superscript lowercase letters in the same row indicate the presence of statistically significant differences, *p* < 0.05. * Data analyzed with the Mann–Whitney U test, ** data analyzed with the Friedman’s test and Dunn’s post hoc test.

**Table 3:** Characterization of the newborn.

Parameter	Median (Q1–Q3)
Birth weight (g)	3200 (3090–3580)
Birth size (cm)	50.5 (49.6–51.4)
Head circumference (cm)	34.3 (33.5–35.4)
**Microbiota**	**Median (Q1–Q3)**
Bacteroidetes (mm^3^)	36.3 (32.8–71.2)
Firmicutes (mm^3^)	12.8 (7.5–57.8)
Ratio F/B	0.43 (0.21–0.65)

**Table 4:** Composition of the intestinal microbiota of the mother-newborn pair.

Parameter	Maternal Median (Q1–Q3)	Newborn Median (Q1–Q3)	*p*
Bacteroidetes (mm^3^)	39.8 (33.0–69.8)	36.3 (32.8–71.2)	0.2820
Firmicutes (mm^3^)	15.4 (10.4–66.7)	12.8 (7.5–57.8)	0.1652
Ratio F/B	0.44 (0.27–0.67)	0.43 (0.21–0.65)	0.3145

*n* = 89. Data analyzed with the Mann–Whitney U test.

**Table 5:** Correlations between *Bacteroidetes*, *Firmicutes,* and the F/B ratio with maternal variables assessed at the end of pregnancy.

	Weight	BMI	Glucose	Total Cholesterol	Triglycerides	c-HDL	Systolic Pressure	Diastolic Pressure
Bacteroidetes	r = −0.1758 *p* = 0.0994	r = −0.1639 *p* = 0.9869	r = 0.08854 *p* = 0.4093	r = 0.1436 *p* = 0.1794	r = −0.1568 *p* = 0.1424	r = −0.1402 *p* = 0.1899	r = 0.0017 *p* = 0.9869	r = 0.07828 *p* = 0.4659
Firmicutes	r = −0.1666 *p* = 0.1188	r = −0.1070 *p* = 0.3182	r = 0.07897 *p* = 0.4620	r = 0.3676 *p* = 0.0004	r = 0.1374 *p* = 0.1993	r = −0.3302 *p* = 0.0016	r = 0.1320 *p* = 0.2174	r = 0.2598 *p* = 0.0140
Ratio F/B	r = −0.1735 *p* = 0.1040	r = −0.0755 *p* = 0.4814	r = 0.07341 *p* = 0.4942	r = 0.3490 *p* = 0.0008	r = 0.2137 *p* = 0.0444	r = −0.3014 *p* = 0.0041	r = 0.2597 *p* = 0.0140	r = 0.2999 *p* = 0.0043

*n* = 89. Data was analyzed with the Spearman test. Statistically significant difference at *p* < 0.05.

**Table 6:** Correlations between maternal *Bacteriodetes*, *Firmicutes*, and F/B ratio with fetal variables evaluated at different times during pregnancy.

	Bacteroidetes Correlation	Firmicutes Correlation	Ratio F/B		Bacteroidetes Correlation	Firmicutes Correlation	Ratio F/B
**Fetal Variable Evaluated**				**Fetal Variable Evaluated**			
LCC 1	r = 0.4005 *p* = 0.0001	r = 0.2715 *p* = 0.0101	r = 0.2003 *p* = 0.0598	FL 2	r = 0.1606 *p* = 0.1328	r = 0.2546 *p* = 0.0161	r = 0.1694 *p* = 0.1126
LCC 2	r = 0.3055 *p* = 0.0036	r = 0.0964 *p* = 0.3686	r = −0.0209 *p* = 0.8452	FL 3	r = 0.3417 *p* = 0.0010	r = 0.2701 *p* = 0.0105	r = 0.1016 *p* = 0.3433
DBP 2	r = 0.1683 *p* = 0.1148	r = 0.1557 *p* = 0.1450	r = 0.0658 *p* = 0.5400	FL 4	r = −0.2310 *p* = 0.0294	r = −0.4132 *p* < 0.0001	r = −03658 *p* = 0.0004
DBP 3	r = 0.1880 *p* = 0.0776	r = 0.0916 *p* = 0.3929	r = −0.1057 *p* = 0.3244	FCF 2	r = 0.0466 *p* = 0.6643	r = 0.0770 *p* = 0.4733	r = −0.0414 *p* = 0.6995
DBP 4	r = 0.1020 *p* = 0.3414	r = −0.1321 *p* = 0.2170	r = −0.2288 *p* = 0.0311	FCF 3	r = −0.04302 *p* = 0.6890	r = −0.0497 *p* = 0.6432	r = −0.0755 *p* = 0.4819
CC 2	r = 0.0818 *p* = 0.4457	r = −0.0117 *p* = 0.9130	r = −0.0440 *p* = 0.6817	FCF 4	r = 0.07917 *p* = 0.4608	r = 0.04365 *p* = 0.6846	r = −0.0308 *p* = 0.7742
CC 3	r = 0.1735 *p* = 0.1039	r = 0.2278 *p* = 0.0318	r = 0.1023 *p* = 0.3402	DV 2	r = 0.2033 *p* = 0.0560	r = 0.2328 *p* = 0.0281	r = 0.0827 *p* = 0.4408
CC 4	r = 0.0070 *p* = 0.9479	r = −0.0920 *p* = 0.3907	r = −0.2100 *p* = 0.0482	DV 3	r = 0.2008 *p* = 0.0592	r = 0.2754 *p* = 0.0090	r = 0.2272 *p* = 0.0323
AC 2	r = 0.1443 *p* = 0.1772	r = 0.2623 *p* = 0.0130	r = 0.1721 *p* = 0.1068	PE 3	r = 0.4921 *p* < 0.0001	r = 0.4653 *p* < 0.0001	r = 0.2344 *p* = 0.0271
AC 3	r = 0.2854 *p* = 0.0067	r = 0.4029 *p* < 0.0001	r = 0.2345 *p* = 0.0270	PE 3	r = −0.1108 *p* = 0.3014	r = −0.2890 *p* = 0.0060	r = −0.3635 *p* = 0.0005
AC 4	r = −0.121 *p* = 0.2545	r = −0.3630 *p* = 0.0005	r = −0.3752 *p* = 0.0003				

*n* = 89. Data analyzed with the Spearman test.

## Data Availability

All data generated or analyzed during this study are included in this published article. The datasets generated during and/or analyzed during the current study are available from the corresponding author upon reasonable request.

## Abbreviations

The following abbreviations are used in this manuscript:

TC	Total cholesterol
TG	Triglycerides
cHDL	High-density lipoprotein cholesterol
